# Cross‐Scale Synergistic Optimization for Miniature Meta‐Liquid Hybrid Zoom Imaging

**DOI:** 10.1002/nap2.70185

**Published:** 2026-07-01

**Authors:** Zhuoxuan Fan, Meng Zhang, Zihao Gan, Mengyue Chang, Ce Zhang, Zhaoyang Liu, Jianming Lv, Kunsheng Xing, Zhuoqun Hu, Junbo Yang, Huajie Hong

**Affiliations:** ^1^ College of Intelligence Science and Technology National University of Defense Technology Changsha China; ^2^ National Key Laboratory of Equipment State Sensing and Smart Support National University of Defense Technology Changsha China; ^3^ School of Optics and Photonics Beijing Institute of Technology Beijing China; ^4^ College of Science National University of Defense Technology Changsha China

## Abstract

Miniaturized zoom imagers often face a three‐way trade‐off among tunability, chromatic correction, and optical length. Here, we propose a cross‐scale synergistic optimization framework for a metasurface‐liquid lens hybrid imager, in which electrowetting actuation, system‐level aberration correction, and meta‐atom phase, group‐delay (GD), and fabrication constraints are optimized in a unified design space. The resulting system uses two liquid lenses, two spherical refractive lenses, and one achromatic metalens to realize 1.5× continuous visible‐band zoom over a 4–6 mm focal‐length range, a 10° field of view, and a 15.5 mm total optical length. Rather than acting as an isolated focusing element, the metalens serves as a compact dispersion and high‐order aberration modulator embedded in the full objective. Full‐system wave‐optics simulations show that boundary–phase optimization improves multi‐wavelength focal consistency while exposing the residual halo imposed by phase folding. Etch‐height and system‐level tolerance analyses further indicate practical manufacturability margins for the hybrid design.

## Graphical abstract

A physically constrained optimization framework links electrowetting liquid lenses and metasurface dispersion engineering to realize a compact visible‐band achromatic zoom imager. By co‐optimizing zoom actuation, meta‐atom manufacturability, and system aberrations, the design achieves 1.5x zoom in a 15.5 mm track and clarifies robustness against fabrication and alignment errors.

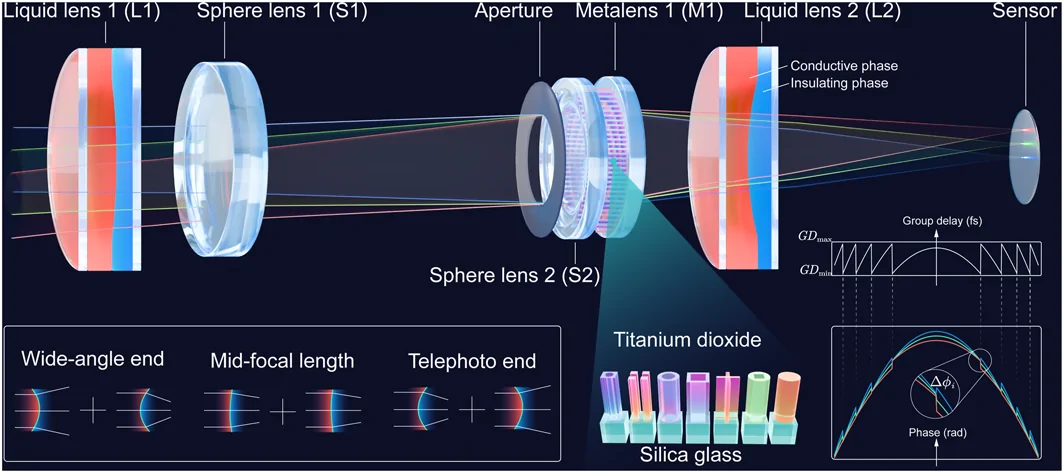

## 1 Introduction

Liquid lens technology based on electrowetting on dielectric (EWOD) offers a non‐mechanical biomimetic solution for dynamic tuning [1]. Its working mechanism involves modulating the curvature of a liquid–liquid interface by altering the wettability of the solid‐liquid interface via an external voltage, thus continuously varying the optical power. This absence of moving mechanical parts has shown immense potential in biomedical imaging [2], adaptive optics [3], and augmented reality displays [4, 5]. With the optimization of autofocus algorithms [6], the maturation of large‐aperture designs [7], and the multifunctionalization of control mechanisms [8], the application scope of liquid lenses continues to expand. However, liquid lenses still face physical bottlenecks in high‐performance imaging: the refractive index difference (Δn) between the two liquids is typically much lower than that of glass–air interfaces, and the dynamic range of interface curvature is limited by saturation voltage. Consequently, the tunable optical power range of a single liquid lens is often insufficient. Conventional solutions typically cascade multiple solid and liquid lenses and employ computational optics for aberration correction [9, 10], which inevitably leads to increased system volume and complexity.

To overcome the bulkiness of traditional correction methods, optical metasurfaces mark a paradigm shift in electromagnetic wave manipulation. By leveraging subwavelength structural units, metasurfaces have demonstrated the capability for arbitrary control over wavefront amplitude, phase, and polarization [11], laying the foundation for next‐generation ultra‐compact high‐performance optical systems. Although multifunctional multiplexing strategies have emerged to decouple degrees of freedom such as wavelength [12], polarization [13, 14], amplitude [15], or incident angle [16, 17], they often necessitate compromises in efficiency due to channel crosstalk [18]. Furthermore, conventional metasurfaces are typically static devices; their optical functions are fixed upon fabrication. This intrinsic “write‐once” characteristic severely restricts their adaptability and flexibility in dynamic scenarios [19]. Although researchers have attempted to endow metasurfaces with dynamic tunability using active materials [20, 21, 22, 23, 24] or mechanical displacement [25, 26, 27, 28], these approaches often introduce significant hurdles such as high optical loss, fabrication complexity, or reliance on bulky mechanical actuators. Therefore, integrating static high‐performance metasurfaces with non‐mechanical liquid lenses represents a theoretically ideal hybrid architecture: The metasurfaces provide strong wavefront shaping capabilities to correct the aberrations of the liquid lenses, whereas the liquid lenses provide the dynamic zooming that static metasurfaces lack.

Despite their rapid independent development, synergistic design deeply fusing these heterogeneous mechanisms remains unexplored. Existing “refractive‐meta” hybrid systems often adopt a decoupled strategy [29, 30, 31, 32]: a macroscopic system is designed based on geometric optics first, followed by phase extraction for metasurface design. This unidirectional serial workflow severs the coupling between macroscopic optical performance and the physical limits of nanostructures (e.g., local phase gradient and maximum group delay). Consequently, when facing complex regulation demands such as large bandwidths or wide fields of view (FOV), designs often fail due to the physical unrealizability of the required metasurface.

Addressing these challenges, we propose a metasurface–liquid lens hybrid zoom imaging system based on cross‐scale synergistic optimization, as shown in Figure 1. The system consists of two electrowetting liquid lenses for zooming and image‐plane compensation, two spherical refractive lenses for optical‐power distribution and low‐order aberration correction, and an achromatic metalens for dispersion and high‐order aberration modulation. The key idea is not to simply stack a static metalens onto a liquid‐lens system, but to simultaneously handle macroscopic optical parameters, liquid‐lens electrowetting responses, and meta‐atom phase/group‐delay/fabrication constraints within the same optimization solution space. With this strategy, the system realizes a 1.5× continuous zoom over 4–6 mm and a 10° field of view within a total optical length of 15.5 mm. Its engineering feasibility is further examined through constraint–convergence visualization, tolerance analysis, purely refractive baselines, and full‐system wave‐optics simulation.

**FIGURE 1:**
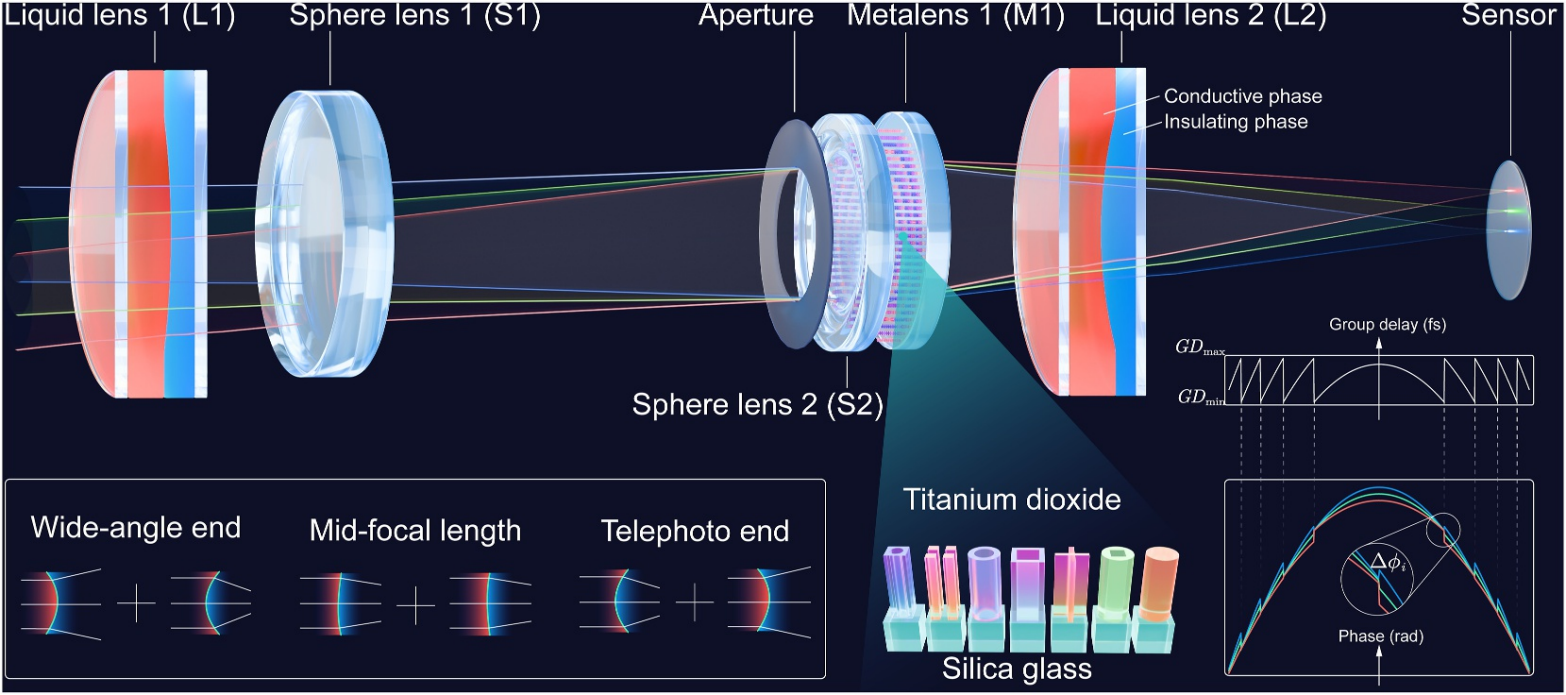
Schematic architecture of the meta‐liquid hybrid zoom imaging system. The system consists of a front liquid lens (L1), a first spherical lens (S1), an aperture stop, an achromatic metalens (M1), a second spherical lens (S2), and a rear liquid lens (L2), arranged sequentially along the optical axis. Based on the electrowetting effect, the liquid lenses continuously adjust optical power by voltage‐tuning the curvature of the interface between the conductive and insulating phases. L1 and L2 synergistically perform the zooming and focusing functions. The metalens M1 employs multi‐zone dispersion engineering, utilizing group delay folding and optimizing phase interference at zone boundaries to achieve broadband achromaticity and aberration correction across the visible spectrum.

## 2 Results

### 2.1 Design Principles

The zooming and focusing capabilities of the hybrid imaging system rely on electrowetting‐based liquid lenses. The physical response of the liquid interface is governed by the Young–Lippmann equation [7]. Fundamentally, the contact angle θ of the conductive liquid on the dielectric surface changes with the applied voltage U:

(1) $$\cos\;\theta =\cos\theta_{0}+\frac{\varepsilon_{r}\varepsilon_{0}}{2\gamma_{ci}d}U^{2}$$

where $\theta_{0}$ is the initial contact angle, $\varepsilon_{r}\varepsilon_{0}$ is the effective permittivity of the dielectric layer, d is the layer thickness, and $\gamma_{ci}$ is the interfacial tension. Because the curvature κ of the liquid–liquid interface is geometrically determined by the contact angle and the aperture radius R, the relationship converting the control voltage to the curvature can be derived as follows:

(2) $$\kappa =\kappa_{0}+\frac{\varepsilon_{r}\varepsilon_{0}}{2\gamma_{ci}dR}U^{2}$$

where $\kappa_{0}$ is the initial interface curvature at zero voltage, $\varepsilon_{r}$ is the combined relative permittivity of the dielectric and hydrophobic layers, $\varepsilon_{0}$ is the vacuum permittivity, $\gamma_{ci}$ is the interfacial tension between the conductive and insulating phases, d is the dielectric layer thickness, and R is the aperture radius of the liquid lens. Figure 2 illustrates the dependency of interface curvature, conductive phase thickness, and oil bodycone thickness on the applied voltage for the liquid lens used in this study. As the voltage increases from 31 to 64 V, the liquid lens transitions from negative to positive optical power following a quadratic function with a slight initial curvature. Concurrently, governed by the principle of mass conservation, the thicknesses of the conductive phase and the oil bodycone decrease monotonically.

**FIGURE 2:**
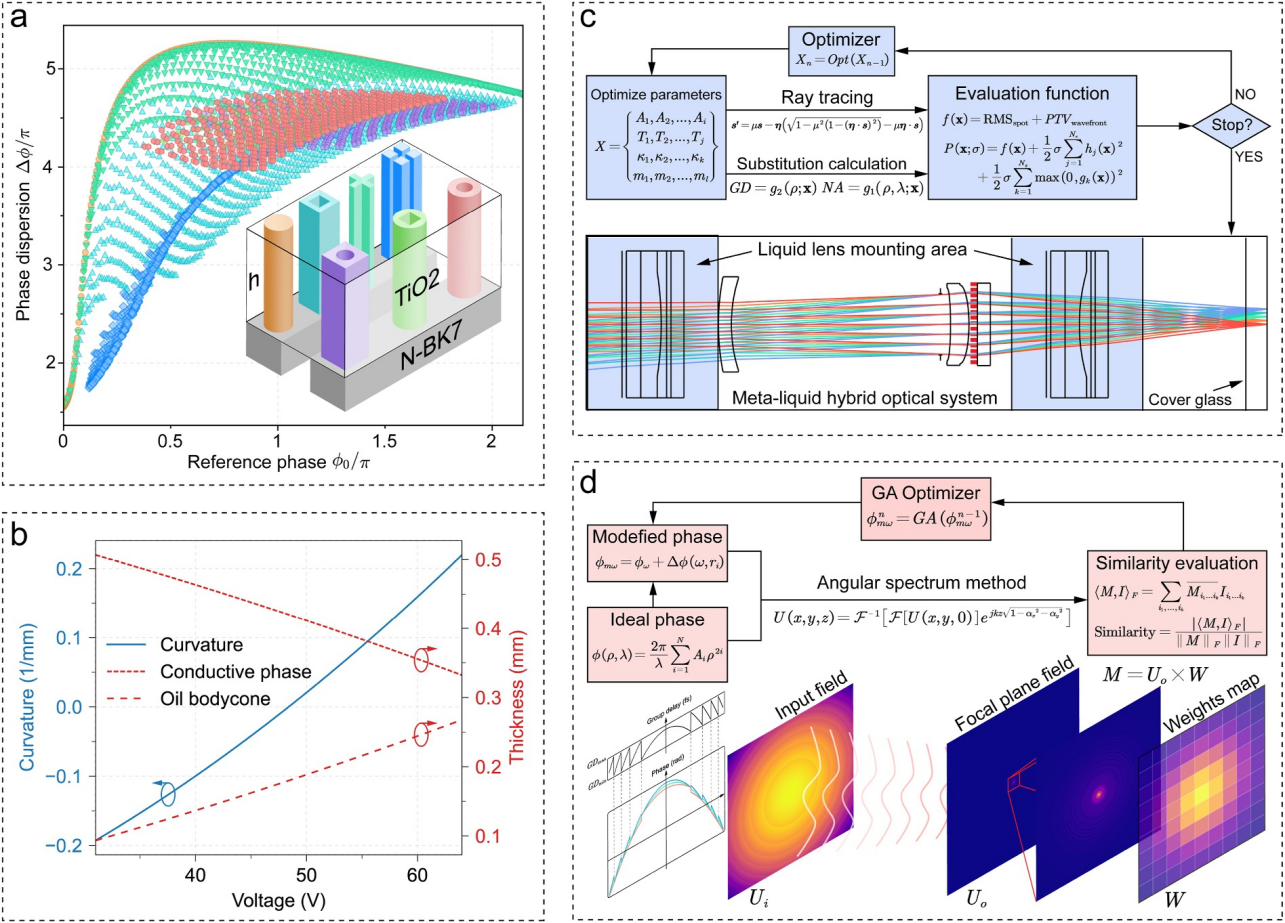
Algorithmic framework of the meta‐liquid hybrid imaging system. (a) Phase‐dispersion space map calculated at λ=700 nm for seven distinct meta‐atom cross‐sections using the waveguide effective refractive index method. The meta‐atoms consist of ${\text{TiO}}_{2}$ nanopillars (h=650 nm) on an N‐BK7 substrate. (b) Relationship between the applied voltage and the curvature of the conductive/insulating phase interface (left axis), and the variation in thickness of the conductive phase and the oil cone (right axis). (c) Block diagram of the hybrid optimization algorithm and the resulting optimized system configuration. (d) Block diagram of the multi‐zone dispersion engineering algorithm for the achromatic metalens, illustrating the propagation of the group‐delay‐folded phase in free space.

For the achromatic metalens design, a phase model based on even‐order radial polynomials [31] is adopted. This model is widely used for its ability to precisely describe complex axisymmetric wavefronts. The phase distribution is defined as a function of the wavelength‐independent part of the optical path difference (OPD) and the wavelength‐dependent part:

(3) $$\phi \left(\rho ,\lambda \right)=\frac{2\pi }{\lambda }\sum_{i=1}^{N}A_{i}\rho^{2i}$$

where ϕ(ρ,λ) is the phase at radius ρ and wavelength λ, and $A_{i}$ are the polynomial coefficients. Crucially, this formulation not only defines the requisite spatial phase profile (e.g., for focusing or aberration correction) but also establishes a dispersion relationship that aligns closely with the intrinsic dispersion response of meta‐atoms based on first‐order effective medium theory [33]. This alignment facilitates broadband phase matching. In contrast, although exploiting strong resonances in meta‐atoms can introduce non‐linear dispersion for higher design degrees of freedom, it often results in reduced efficiency, limited bandwidth, and drastically increased design complexity [34]. Therefore, this study employs the design phase described by Equation (3) to fully leverage the natural dispersion characteristics of the metasurface structures, ensuring high efficiency across a broadband range while minimizing design difficulty.

In the design of hybrid meta‐optical systems, traditional optimization workflows exhibit significant intrinsic limitations. Conventional optical design typically relies on damped least squares (DLS) or meta‐heuristic algorithms, which focus primarily on minimizing image quality merit functions (e.g., RMS spot radius or wavefront error) [35]. However, such strategies face severe challenges when dealing with complex hybrid systems comprising liquid lenses and metasurfaces. Because these methods fail to couple macroscopic optical performance with microscopic device physics, algorithms tend to converge toward “mathematically optimal but physically infeasible” solutions—results that violate the voltage‐curvature response of liquid lenses or the local fabrication limits of metasurfaces, rendering them unrealizable in engineering practice.

To overcome this challenge, we propose a synergistic optimization framework based on the quadratic penalty function method. This framework not only targets imaging quality but also explicitly maps the “electro‐fluidic‐geometric” model of the liquid lenses and the “phase‐dispersion‐fabrication” constraints of the metasurface into the optimization solution space. To make the cross‐scale optimization procedure explicit, the optimization variables, constraint types, and monitored quantities are defined as follows. The system‐level variables include liquid‐lens driving voltages, liquid–liquid interface curvatures, solid‐lens curvatures/thicknesses, air gaps, and the even‐order polynomial phase coefficients of the single static metasurface. The system‐level monitored outputs include the EFL, RMS spot radius, and MTF at each zoom state, whereas the metasurface monitored quantities are the full‐aperture maximum local phase gradient and maximum GD of the same static metalens. The voltage–curvature relation of the liquid lenses is treated as an equality constraint $h_{j}\left(x\right)$, whereas the local phase‐gradient and GD limits of the metasurface are treated as inequality constraints $g_{k}\left(x\right)$. An augmented objective function P(x;σ) is defined to transform the original constrained nonlinear optimization problem into a sequence of unconstrained sub‐problems for iterative solution:

(4) $$P\left(x;\sigma \right)=f\left(x\right)+\frac{1}{2}\sigma \sum_{j=1}^{N_{e}}h_{j}{\left(x\right)}^{2}+\frac{1}{2}\sigma \sum_{k=1}^{N_{g}}\max{\left(0,g_{k}\left(x\right)\right)}^{2}$$

where x represents the set of optimization parameters, and σ is the penalty coefficient for equality and inequality constraints. f(x) is the merit function for imaging quality. $h_{j}\left(x\right)$ represents the equality constraints, which force the optimization path to strictly adhere to the physical actuation model of the liquid lens, ensuring that every zoom configuration corresponds to a realistic, tunable voltage value. $g_{k}\left(x\right)$ represents the inequality constraints, designed to delimit the physically feasible domain of the metasurface. By penalizing solutions that exceed local numerical aperture (NA) limits or maximum group delay capacities, these constraints ensure the manufacturability of the device under current lithography processes and the validity of dispersion control. The specific forms of $h_{j}\left(x\right)$ and $g_{k}\left(x\right)$ are detailed in Appendix A. By internalizing physical constraints as numerical barriers via the potential function, this method enables the conversion of constrained problems into unconstrained ones, serving as the core engine for realizing the design of the meta‐liquid hybrid optical system.

Figure 3 visualizes the evolution of two key classes of physical constraints during optimization. For the metasurface, the plotted GD and phase‐gradient values are full‐aperture maxima of the same static metalens, whereas the wide, mid, and tele curves represent how the system‐level performance at different zoom states is projected onto this shared metasurface constraint space during joint optimization. As the penalty terms participate in the optimization, these monitored projections move toward the feasible domain bounded by GD≤130 fs and the phase‐gradient constraint. The final checkpoint is located near the edge of this feasible domain, indicating that the system makes full use of the dispersion and phase‐modulation capability of the metasurface without crossing the manufacturability boundary. For the liquid lens, the voltage and interface curvature are not arbitrary independent variables but must lie on the electrowetting response surface. The optimization trajectory approaches this feasible surface from outside the physical model, showing that each zoom state is optimized not only in the sense of geometrical aberration correction but also as an actually drivable liquid‐lens state.

**FIGURE 3:**
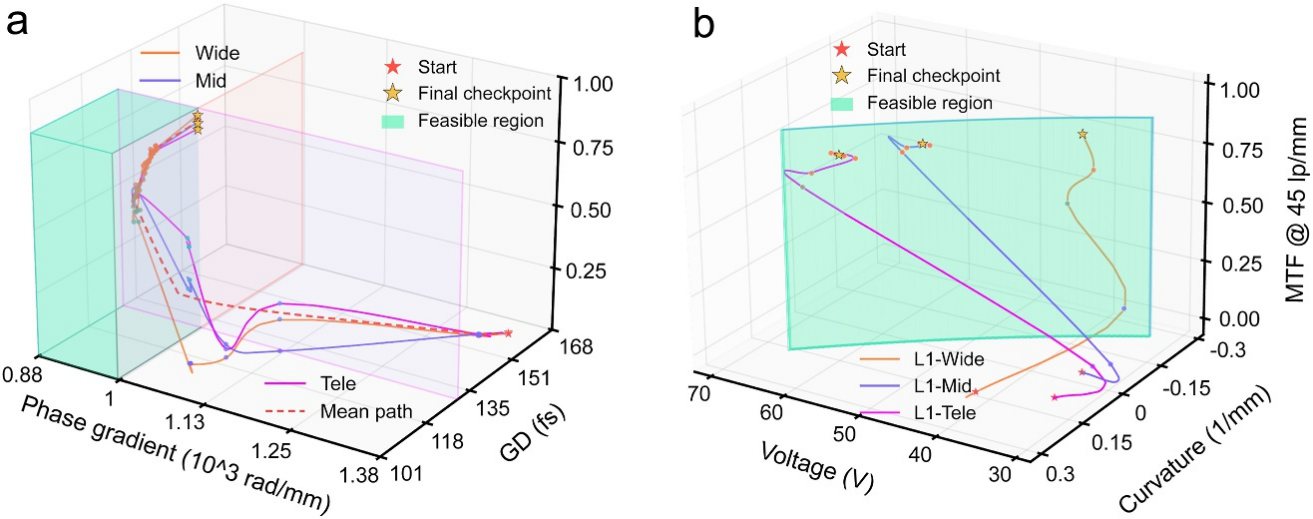
Cross‐scale constraint convergence during optimization. (a) Optimization trajectories in the metasurface constraint space, where the horizontal axes represent the full‐aperture maximum phase gradient and maximum GD of the same static metalens, and the vertical axis represents the monitored MTF at 45 lp/mm. The translucent constraint surfaces indicate the inequality boundaries for phase gradient and GD, whereas the light‐green volume denotes the feasible domain. The three trajectories correspond to wide‐, mid‐, and tele‐state performance projections of the joint system optimization onto the shared metasurface constraint space, and the red averaged trajectory indicates the overall evolution of the joint optimization. (b) Equality‐constraint trajectory for the voltage‐curvature relation of liquid lens L1. The translucent surface represents the feasible voltage–curvature relation derived from the electrowetting model; the optimization points progressively converge from outside the model surface into the tolerance band and continue to improve the imaging merit function near the feasible manifold.

### 2.2 Ray Optics

The ray‐optics based optimization workflow is illustrated in Figure 2c. The process begins by defining the set of optical parameters to be optimized. Key decision variables include the polynomial coefficients $A_{i}$ of the metalens OPD function, the center thicknesses $T_{j}$ of all optical elements and air gaps, the interface curvatures $\kappa_{k}$ of the solid and liquid lenses, and the refractive indices $m_{l}$ of the solid lenses. Subsequently, ray tracing algorithms simulate beam propagation from the object plane to the image plane. By analyzing ray intercepts and optical path accumulation at the image plane, system performance metrics including root mean square (RMS) spot radius, peak‐to‐valley (PTV) wavefront error, and effective focal length (EFL) are computed.

Distinct from conventional optical design flows, this study integrates physical constraints directly into the optimization loop. Beyond calculating merit functions based on statistical ray tracing data, the algorithm automatically extracts the even‐order polynomial coefficients during each iteration to compute the local numerical aperture (NA) and group delay (GD) of the metasurface (detailed calculation methods are provided in Appendix A). These values are cross‐referenced against the modulation limits of a pre‐constructed meta‐atom library (Figure 2a). Rigorous penalty terms are imposed within the objective function to strictly constrain the phase modulation degrees of freedom, thereby ensuring manufacturability. Finally, a composite objective function, synthesizing imaging quality metrics and physical constraint penalties, is iteratively minimized using damped least squares (DLS) and global heuristic algorithms across multiple configurations.

The optimized architecture of the meta‐liquid hybrid zoom system is depicted in Figure 2c. The system adopts a symmetric layout, with two liquid lenses positioned at the front (L1) and rear (L2), and an aperture stop placed centrally to balance off‐axis aberrations. A thin solid lens is introduced between L1 and the stop to compensate for primary spherical aberration generated by L1 and to synergistically correct coma with the solid lens behind the stop. The post‐stop solid lens undertakes field‐dependent coma correction and compensation for spherical aberration introduced by L2. Crucially, it shares a portion of the optical power, thereby alleviating the phase gradient requirements on the metalens. The metalens is strategically placed behind the stop to restrict the range of incident angles, effectively suppressing angular dispersion [16] and the resulting high‐order aberrations associated with large incidence angles [36]. In this architecture, the metalens is primarily responsible for sub‐wavelength wavefront shaping to finely tune low‐order aberrations and balance high‐order terms while also contributing to the optical power. The liquid lenses facilitate dynamic focal length tuning and image plane compensation. The system features an entrance pupil diameter (EPD) of 1 mm and a total optical length (TOL) of merely 15.5 mm, achieving a continuous 1.5× zoom ratio across a focal length range of 4–6 mm (detailed parameters in Appendix B). The light blue regions in the figure indicate mechanical clearance for the liquid lenses, accommodating electrode mounts and connections. Without these packaging constraints, the TOL could be further compressed.

The performance evaluation of the ray‐optics‐optimized system is presented in Figure 4. Figure 4a illustrates the mapping between liquid lens drive voltages and system focal length during zooming. L2 undergoes the larger optical‐power modulation and therefore functions as the main variator, whereas L1 mainly compensates image‐plane shift and fine‐tunes residual aberrations. Previous liquid‐lens zoom systems often increase zoom ratio by adding more tunable lenses or longer relay optics [37]. In contrast, the present system deliberately limits the number of liquid lenses to two, and transfers part of the chromatic and high‐order aberration correction burden to the metasurface. Consequently, rather than pursuing absolute superiority in every image‐quality metric, the primary advantage of this system lies in achieving a compact 1.5× visible‐band zoom within a 15.5 mm TOL under a low liquid‐lens count.

**FIGURE 4:**
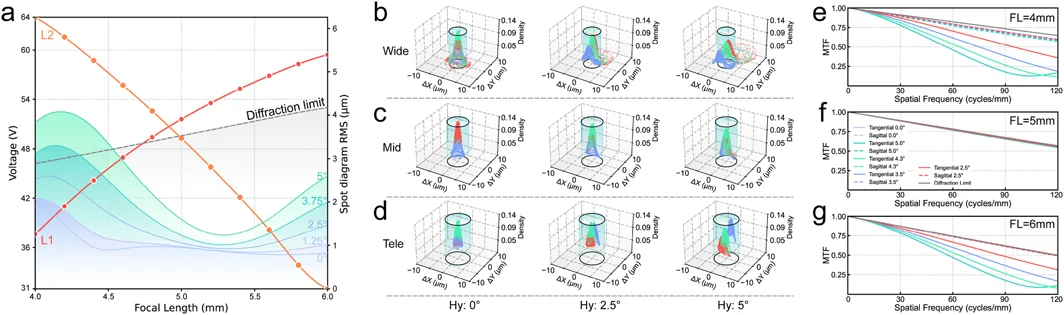
Performance metrics of the designed hybrid optical system. (a) Evolution of system parameters during zooming: the relationship between the drive voltages of L1/L2 and the effective focal length (EFL) (left axis), and the RMS spot radius versus EFL at different fields of view (FOV) (right axis). The gray dashed line indicates the Airy disk radius. (b–d) Ray landing distributions and relative spot densities for the wide‐angle, intermediate, and telephoto states at different incident angles (0° to 5°) and wavelengths (486 nm, 588 nm, and 656 nm). The columnar regions represent the airy disk. (e–g) modulation transfer function (MTF) distributions at the wide‐angle (FL = 4 mm), intermediate (FL = 5 mm), and telephoto (FL = 6 mm) states, respectively; solid lines represent the tangential (meridional) direction, and dashed lines represent the sagittal direction.

The filled regions in Figure 4a (right axis) display the geometrical RMS spot radii across different fields of view (FOV), with the gray dashed line marking the airy‐radius reference. At the ray‐optics level, the optimized system maintains compact spot sizes over most focal lengths and fields. Minor degradation occurs at the edge fields of the short focal length state, which is attributed to the curvature modulation saturation of the liquid lens at extreme voltages. At the short focal end, the demand for optical power surges, forcing the solid lenses and metalens to bear higher loads. This concentrated power distribution deviates somewhat from Hopkins' power distribution principles, leading to increased residual primary aberrations. To address the core challenge of insufficient liquid lens power in short‐focal, high‐NA configurations, the metalens assumes complex wavefront correction tasks, synergizing with standard spherical lenses to compensate for the physical limitations of the liquid components.

Figure 4b details the ray intercept distributions (spot diagrams) at different wavelengths. Here, ΔX and ΔY denote lateral deviations on the image plane, the Z‐axis represents ray density (energy distribution), and the cylindrical region signifies the Airy‐disk reference. For the on‐axis field (0°), the spots for three wavelengths strongly overlap, demonstrating effective axial chromatic aberration correction in the geometrical‐optics model. As the FOV increases, the centroids of the spots gradually separate, most notably at the maximum field, revealing the presence of lateral color. Furthermore, red light energy density slightly exceeds blue light at the short focal end, whereas the reverse trend appears at the long focal end, reflecting the dynamic nature of spherochromatism with focal length. Some energy spillover and peak‐density reduction remain at the maximum field, indicating that the subsequent wave‐optics analysis is necessary to quantify diffraction‐order halo and raw‐contrast limitations.

The geometrical‐optics MTF performance is shown in Figure 4e,f. At the central focal length of 5 mm, the ray‐optics design exhibits high simulated contrast. At the short and long focal extremes, the separation between meridional and sagittal MTF curves indicates residual off‐axis astigmatism and coma. These residuals primarily stem from the intrinsic dispersion of the liquid materials and constraints on optical power distribution, often requiring additional elements or computational imaging algorithms for compensation [9, 10]. The geometrical MTF curves therefore indicate that the cross‐scale optimizer provides a strong system‐level starting point, whereas the full‐system wave‐optics analysis in Figure 5 further quantifies the diffraction‐order halo introduced by the phase‐folded metasurface.

**FIGURE 5:**
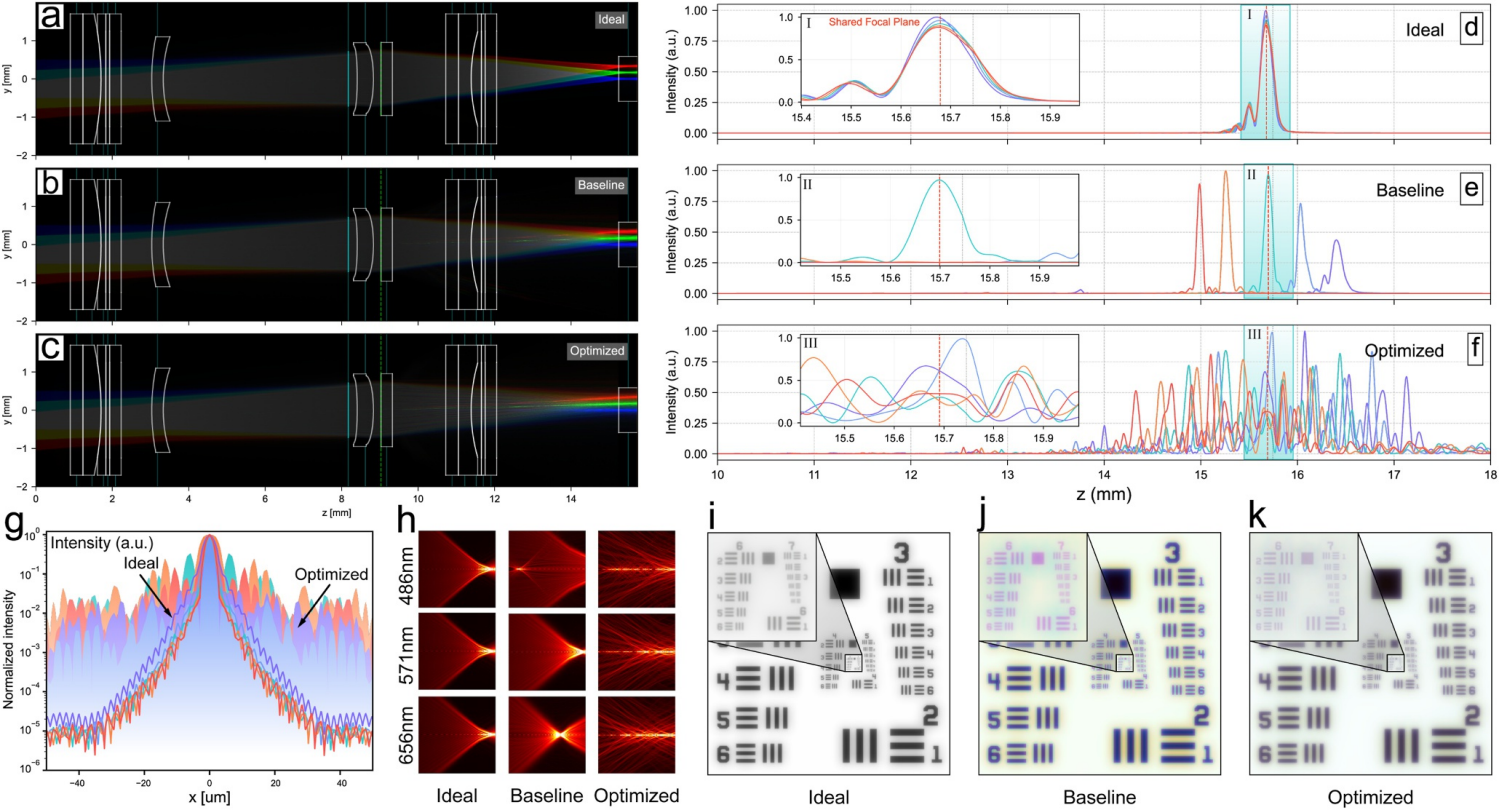
Full‐system wave‐optics simulation results. (a–c) YZ‐plane multi‐wavelength intensity distributions of the complete metasurface–liquid lens hybrid system for the ideal, GD‐folded baseline, and boundary–phase‐optimized phases, displayed with an $I^{0.4}$ gamma correction. The three colors represent different fields of view, and the wireframes indicate the positions of L1, S1, the aperture stop, S2, M1, L2, and the CMOS cover glass. (d–f) Multi‐wavelength on‐axis intensity distributions for the three schemes, used to evaluate longitudinal chromatic aberration and ghost foci in the full system. (g) Multi‐wavelength logarithmic PSF center profiles comparing the full‐precision ideal reference and the optimized system; solid curves denote the ideal reference, and filled profiles denote the optimized result. (h) Matrix of axial logarithmic intensity distributions for the three schemes at 486 nm, 571 nm, and 656 nm, displayed with an $I^{0.4}$ gamma correction. (i–k) RGB simulated images of a USAF‐1951 resolution target for the ideal, baseline, and optimized schemes.

### 2.3 Physical Optics

In the physical implementation of the metasurface, phase coverage, dispersion coverage, transmission efficiency, and manufacturability must be considered simultaneously. Figure 6 show the target phase, phase‐gradient, and GD distributions obtained from the system‐level geometrical‐optics optimization. The nonlinear radial phase‐gradient profile reflects the degrees of freedom used by the metasurface to modulate high‐order aberrations. Figure 6 show that the selected library of 17 ${\text{TiO}}_{2}$ meta‐atoms maintains full 0−2π phase coverage at the representative RGB wavelengths, with average transmittances of approximately 0.76, 0.94, and 0.96 at 486 nm, 588 nm, and 656 nm, respectively. The nominal structural height is set to 650 nm, consistent with the subsequent etch‐height error analysis; this value provides a compromise between GD modulation capability and aspect‐ratio‐related fabrication difficulty.

**FIGURE 6:**
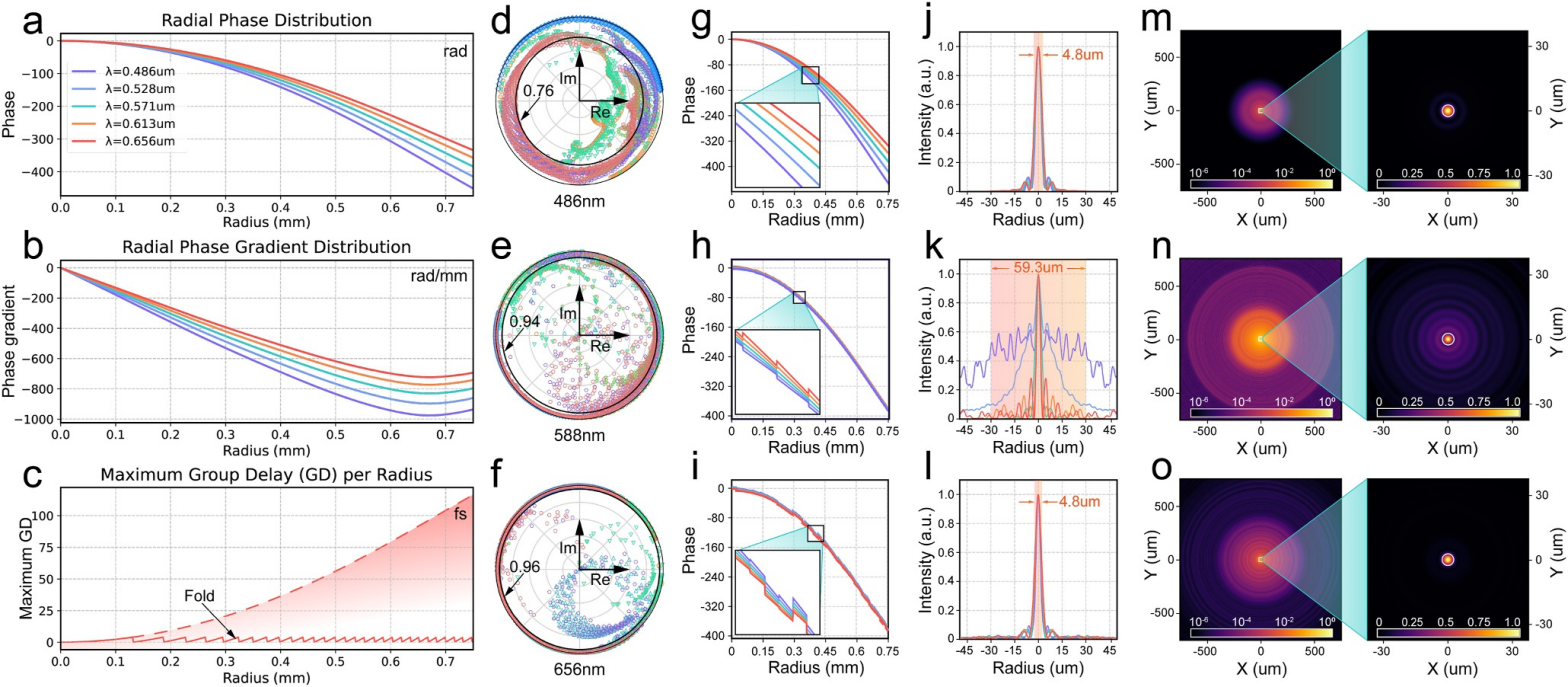
Physical constraints of the metasurface and boundary–phase optimization. (a–c) Optimized phase, phase‐gradient, and GD distributions of the metasurface; the folded equivalent GD is also shown below panel (c). (d–f) Phase coverage and transmittance response of the 17 selected meta‐atoms at 486 nm, 588 nm, and 656 nm, with average transmittances of approximately 0.76, 0.94, and 0.96, respectively. (g–i) Phase distributions for the ideal phase, the GD‐folded baseline phase, and the phase obtained after further boundary‐interference optimization; the insets show local details near phase‐jump regions. (j–l) PSF cross sections near the target plane for the three phase strategies. The FWHM values of the ideal and optimized phases are both approximately 4.8 μm, whereas the baseline FWHM expands to approximately 59.3 μm. (m–o) Image‐plane intensity distributions for the three strategies, with logarithmic intensity on the left and linear intensity on the right.

Although the synergistic optimization reduces the GD requirement imposed on the metasurface, wavelength‐scale meta‐atoms still cannot provide arbitrarily large continuous GD compensation over a millimeter‐scale aperture [34]. If the whole metasurface is forced to continuously match the ideal achromatic phase, the metalens is often driven to sacrifice part of its optical‐power contribution, which contradicts the purpose of introducing the metasurface to enhance the modulation capability of the system. Therefore, a multi‐zone GD folding strategy is adopted: Local dispersion matching is preserved within each zone, whereas the phase jumps at the zone boundaries are optimized to tune the interference relation among secondary waves from different annular regions at the system image plane [38]. It should be emphasized that this strategy does not treat the single metalens as an independent perfect focusing element, but as a dispersion and high‐order aberration modulator in the hybrid system. Based on this strategy, the image‐plane complex amplitude can be written as follows:

(5) $$U=P\left[\sum_{k=1}^{K}t\left(\omega ,r\right)\exp\left[\frac{j\omega }{c}\sum_{i=1}^{N}A_{i}r^{2i}\right]e^{j\Delta \phi_{k}\left(\omega ,r_{k}\right)}\right]$$

where P is the diffraction propagation operator, t(ω,r) is the complex amplitude transmission of the meta‐atom at angular frequency ω, K is the total number of folded zones, and $\Delta \phi_{k}\left(\omega ,r_{k}\right)$ is the phase jump at the boundary of the k‐th zone. To maintain invariant relative dispersion within each zone, the phase jump at any frequency ω must satisfy the following dispersion constraint:

(6) $$\Delta_{k}\phi \left(\omega ,r_{k}\right)=\Delta_{k}\phi \left(\omega_{0},r_{k}\right)+\frac{\omega_{0}-\omega }{c}\sum_{i=1}^{N}A_{i}r_{k}^{2i}$$

Equation (6) indicates that, without disturbing the relative GD matching within each zone, a frequency‐dependent zone phase bias can be used to tune the constructive and destructive interference among secondary waves from different annular regions. Figure 6 compare three phase strategies: the ideal phase, the baseline phase with only GD folding, and the phase obtained by further optimizing the boundary phase after GD folding. Although the baseline satisfies the local GD constraint, the phase discontinuities at the zone boundaries introduce pronounced multi‐order diffraction and broadband mismatch; consequently, the PSF FWHM expands from approximately 4.8 μm for the ideal phase to approximately 59.3 μm. After boundary‐phase optimization, the main‐lobe width is restored to approximately 4.8 μm, and the logarithmic intensity distribution becomes closer to that of the ideal phase. This behavior indicates that boundary–phase optimization improves the broadband interference consistency among multi‐zone secondary waves at the system target plane, whereas residual sidelobe and stray energy remain as a cost of phase folding.

Figure 5 further shifts the evaluation object from a single metalens to the complete metasurface–liquid lens hybrid imaging system. Figure 5 show the multi‐wavelength field propagation through the full system for the ideal phase, the baseline phase with only GD folding, and the optimized boundary–phase design. In this calculation, the complex fields at the selected RGB wavelengths are sequentially transformed by the liquid and solid refractive surfaces, aperture stop, metasurface phase, and image‐plane cover glass, and are propagated between elements according to the optimized system spacing. The resulting fields are sampled around the image plane to form the axial YZ maps, on‐axis intensity traces, PSF profiles, and RGB target images. Unlike the isolated analysis of a plane wave incident on a standalone metalens, this simulation therefore better represents the final imaging condition of the hybrid objective.

Figure 5d–f,h show that the GD‐folded baseline forms a well‐aligned focus at the design wavelength of 571 nm, but exhibits clear axial focal shifts and multi‐order diffraction energy peaks at shorter and longer wavelengths, corresponding to severe longitudinal chromatic aberration and ghost foci. After boundary‐phase optimization, the multi‐wavelength energy is redistributed toward a more common focal region near the image plane, indicating that the optimization improves multi‐wavelength interference consistency among different wavelengths and annular zones in the complete optical system. Meanwhile, Figure 5g shows that the optimized system still contains stronger sidelobe and halo energy than the full‐precision ideal reference. The improvement should therefore be understood as system‐level mitigation of the multi‐wavelength mismatch in the GD‐folded baseline under manufacturable phase‐folding constraints, rather than recovery of an ideal diffraction‐limited response. The final USAF‐1951 simulated images further show that the optimized system retains RGB image details more effectively than the GD‐folded baseline, although residual halo reduces raw image contrast.

### 2.4 Robustness, Manufacturability, and Refractive Baselines

To evaluate whether the optimized solution remains compatible with realistic fabrication and alignment errors, manufacturability is examined through two levels of numerical tolerance analysis. The first level focuses on metasurface etch‐height errors Figure 7. Taking the nominal height of 650 nm as the reference, truncated Gaussian height errors of ±5% and ±10% are considered, where the truncation boundary corresponds to ±3σ. Under a first‐order waveguide‐mode sensitivity approximation, height errors perturb both the phase and GD of the meta‐atoms. The Monte Carlo results show that the errors do not monotonically compress the GD coverage. Under the 5% error condition, 136 out of 256 trials show a broadened GD span and 120 show a narrowed span; under the 10% condition, 174 trials broaden and 82 narrow. This indicates that random etch errors mainly perturb the distribution of phase‐GD coverage, rather than simply reducing the dispersion‐control capability.

**FIGURE 7:**
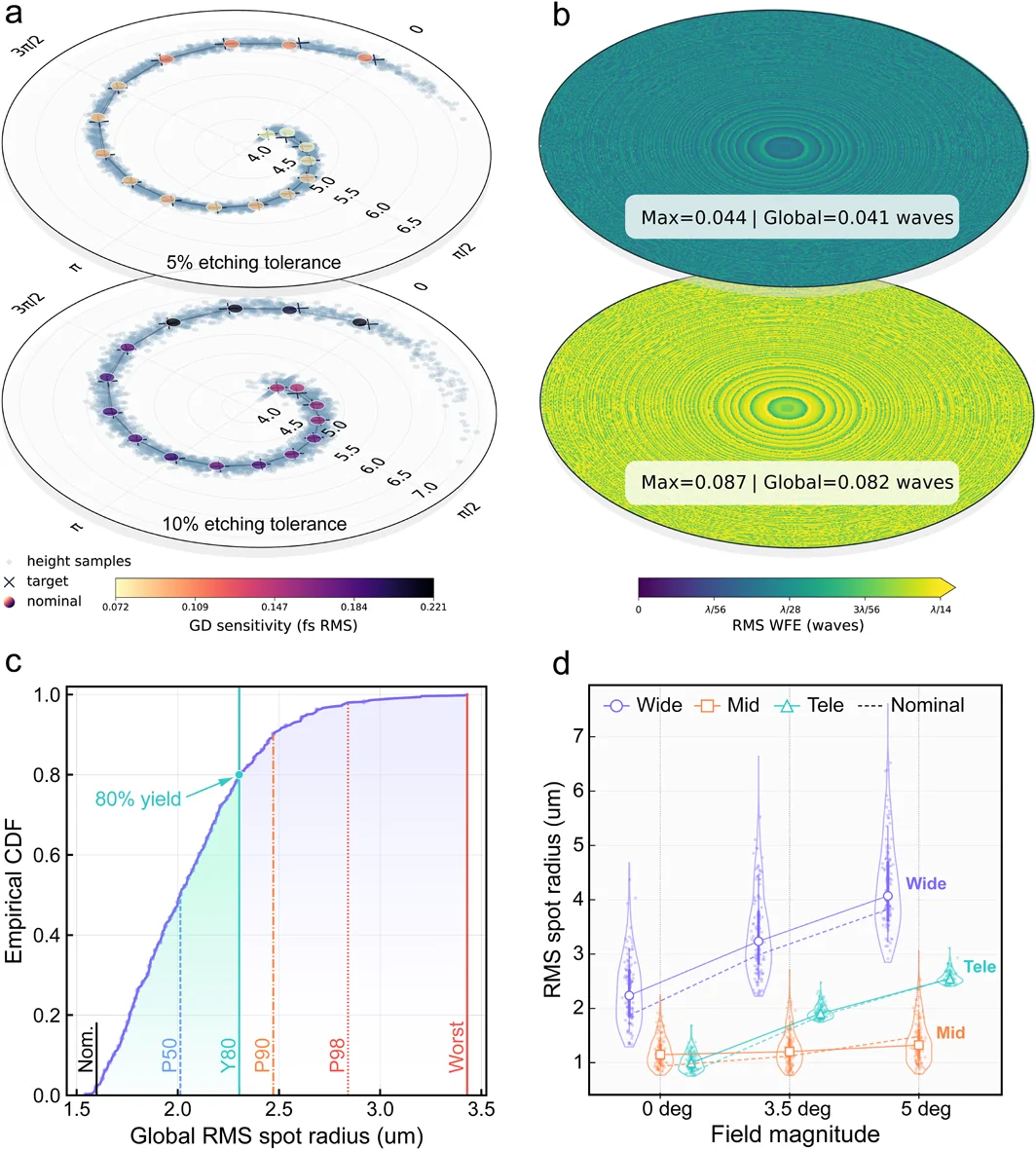
Manufacturing and alignment robustness analysis. (a) Phase‐GD coverage variation of the 17 quantized meta‐atoms under 5% and 10% etch‐height errors, with the scatter‐point color representing GD sensitivity. (b) RMS WFE residual distributions after mapping random height errors onto the full metasurface; the color scale uses λ/14 as the Maréchal criterion reference. (c) Empirical CDF of the global RMS spot radius obtained from Zemax Monte Carlo tolerance analysis, with the arrow marking the 80% yield point. (d) Field‐resolved RMS spot radius distributions at 0°, 3.5°, and 5° fields for the wide, mid, and tele zoom states. Dashed lines denote the nominal design values.

After mapping meta‐atom height errors onto the full metasurface phase distribution, the RMS WFE at the design wavelengths is approximately 0.0271–0.0412 waves under the 5% etch‐height error, all below λ/14. The corresponding residual maps give a maximum RMS WFE of 0.044 waves and a global RMS WFE of 0.041 waves. When the 10% error is used as a stress test, the residual maps increase to a maximum RMS WFE of 0.087 waves and a global RMS WFE of 0.082 waves; the short‐wavelength side is more sensitive, and the 486–537 nm region exceeds or approaches λ/14, whereas the longer wavelengths remain within the criterion. Therefore, the 5% error is taken as a more reasonable nominal process assumption, whereas the 10% case is used to reveal the sensitivity boundary at the short‐wavelength side.

The second level considers system‐level manufacturing and alignment errors. In OpticStudio, Q3‐grade manufacturing/alignment tolerances are used globally, and the sensitivity analysis identifies the Y‐decenter of surface 14 in the rear refractive group as a critical term; this local decenter tolerance is therefore tightened to Q2 grade. For 500 Monte Carlo trials, the nominal global RMS spot radius is 1.5985 μm, whereas the Monte Carlo mean, median, P80, P90, and worst‐case values are 2.0603 μm, 2.0130 μm, 2.3038 μm, 2.4719 μm, and 3.4299 μm, respectively, corresponding to an average degradation of approximately 0.4617 μm. The field‐resolved violin plot further indicates that the wide zoom state shows the largest statistical spread and the strongest separation from the nominal design values, whereas the mid and tele states show smaller Monte Carlo deviations, suggesting larger tolerance margins at the middle and long focal‐length states.

For the purely refractive reference comparison in Figure 8, two baselines are constructed under the same liquid‐lens count, zoom states, field of view, and compact‐layout constraints. In the sphere–liquid baseline, the metasurface is replaced by a spherical refractive element and the system is re‐optimized. In the asphere–liquid baseline, the metasurface is replaced by a 16th‐order even‐asphere element and the system is re‐optimized. Catalog‐glass substitution is enabled for the replacement refractive element and the solid refractive groups in both refractive baselines. The optimized glass sets are H‐ZF71/S‐FPL53/QF50 for the sphere–liquid baseline and S‐TIH23/S‐FPL53/QF8 for the asphere–liquid baseline, whereas the hybrid metalens is implemented as ${\text{TiO}}_{2}$ nanopillars patterned on the standard N‐BK7 substrate used in the metasurface design.

**FIGURE 8:**
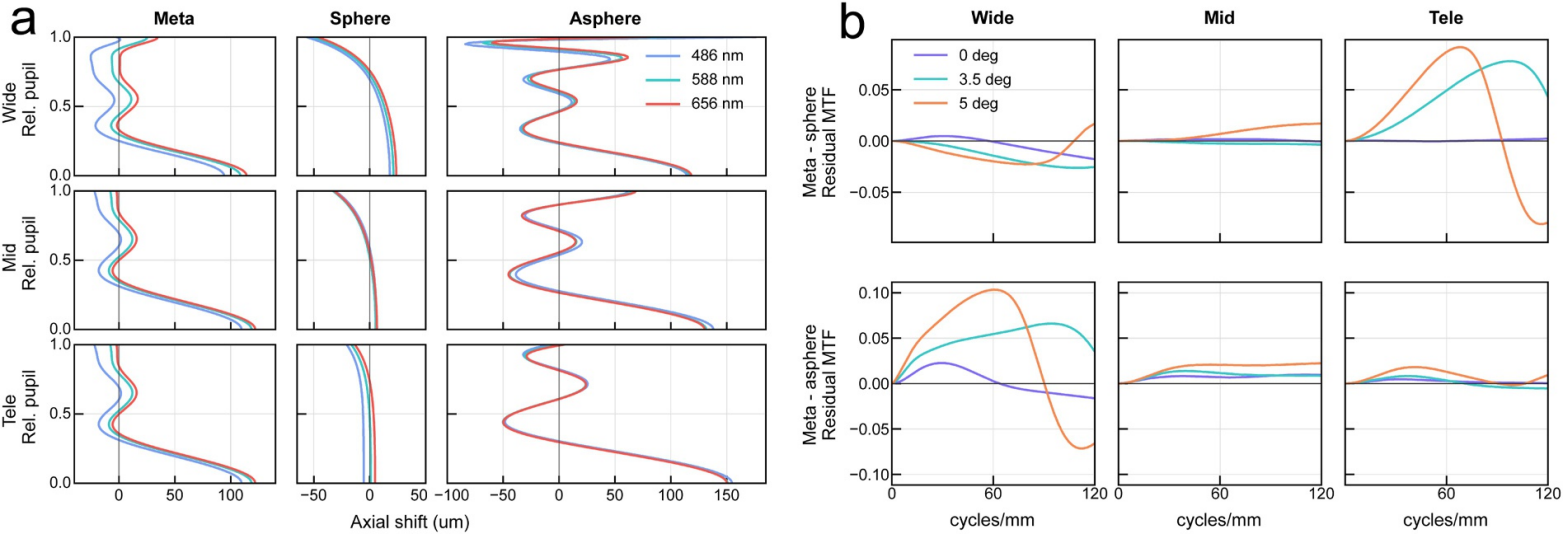
Comparison with refractive liquid‐lens baselines with catalog‐glass substitution enabled. (a) Longitudinal chromatic aberration curves of the meta‐liquid, sphere–liquid, and asphere–liquid systems at the wide, mid, and tele states. The refractive baselines are re‐optimized with catalog‐glass substitution enabled for the replacement refractive element. The optimized glass sets are H‐ZF71/S‐FPL53/QF50 for the sphere baseline and S‐TIH23/S‐FPL53/QF8 for the asphere baseline. The three colors correspond to 486 nm, 588 nm, and 656 nm. (b) MTF residual curves of the meta‐liquid system relative to the sphere–liquid and asphere–liquid baselines. The three columns correspond to the wide, mid, and tele states, and the curve colors denote different fields of view.

These glass distributions provide the refractive references with explicit chromatic‐correction degrees of freedom. In particular, the S‐TIH23/S‐FPL53/QF8 combination in the asphere–liquid baseline combines a high‐index, high‐dispersion glass, an ultra‐low‐dispersion glass well suited to positive optical power, and a medium‐dispersion tuning glass. Together with the even‐asphere surface, this forms a refractive correction channel closer to an apochromatic design strategy than to a simple single‐glass replacement. By contrast, the metalens does not rely on substrate‐glass substitution; its dispersion contribution is introduced through the engineered phase and group‐delay response of the ${\text{TiO}}_{2}$ nanostructures on the fixed N‐BK7 substrate.

Figure 8a plots the resulting longitudinal chromatic‐aberration curves of the meta‐liquid, sphere–liquid, and asphere–liquid systems at the three zoom states. The refractive baselines with catalog‐glass substitution enabled show reduced RGB axial separation through glass selection, especially when the refractive power and chromatic power are distributed across glasses with different dispersion properties. Figure 8b further compares the MTF residuals of the meta‐liquid system relative to the two refractive baselines. These results characterize the different correction routes available within the same compact liquid‐lens layout: refractive glass selection and aspheric geometry in the purely refractive baselines, and fixed‐substrate metasurface phase/GD modulation in the hybrid design.

Table 1 summarizes representative liquid‐lens zoom or tunable imaging systems. Existing systems generally mitigate the limited optical‐power range, chromatic aberration, and dynamic aberration of liquid lenses by increasing the number of liquid elements, extending the refractive relay path, introducing compound‐eye or multi‐channel architectures, or relying on computational restoration. In contrast, the present system uses only two liquid lenses within a total optical length of 15.5 mm, and assigns part of the dispersion and high‐order aberration modulation to a metalens constrained by GD, phase gradient, and etch‐height tolerances. Rather than claiming universal superiority across all image‐quality metrics, this comparison highlights that the meta‐liquid hybrid architecture provides a novel trade‐off among short total length, low liquid‐lens count, and visible‐band achromatic design.

**TABLE 1:** Representative liquid‐lens zoom or tunable imaging systems and this work.

Reference	Application	No axial translation	Tunable liquid elements	Solid/meta elements	Zoom range	TOL/size	Correction strategy
Kuiper et al. (2007) [39]	VGA zoom camera	Yes	2 liquid lenses	3 plastic lenses	1.7×	NR	Special liquids for chromatic correction
Zhao et al. (2018) [40]	Miniature camera objective	Yes, folded path	2 pressure‐tuned aspheric liquid lenses	3 spherical glass lenses	4.5–9 mm, 2×	5 mm × 6.5 mm × 17 mm	Aspheric liquid‐lens aberration control
Yen and Zhang, (2022) [41]	Vehicle ultra‐wide‐angle zoom	Yes	4 liquid‐lens groups	6 spherical lenses	170°/160°/150° FOV states	18 mm total length	Refractive wide‐angle design
Li et al. (2023) [42]	Double‐sided telecentric zoom	Yes	Adaptive liquid lenses	Multi‐element telecentric groups	−0.458× to −0.11×	NR	Refractive telecentric zoom design
Liu et al. (2023) [43]	Adaptive micro‐projection	Yes	Liquid lens	Projection optics	Focus/throw adaptation	NR	Liquid‐lens adaptive projection
Lenk et al. (2022) [44]	Tunable‐lens zoom design method	Conceptual design	Tunable lens groups	First‐order refractive layouts	Design method, not a prototype	NR	Layout search for tunable‐lens zoom systems
Liu et al. (2022) [37]	Continuous zoom microscope	Yes	7 liquid lenses	6 singlets + 2 doublets	10×–60×	About 220 mm length, 25 mm diameter	EDOF and 3D reconstruction algorithms
Zheng et al. (2024) [45]	Adaptive multiscale microscope	Yes	4 liquid lenses	Zoom objective + compound‐eye/sub‐lens array	9×–18×	442 mm optical length	Multiscale optics + distortion correction
Liu et al. (2025) [10]	Compact liquid‐zoom vision system	Yes	3 EWOD liquid lenses	Front/rear spherical groups	28.7–41.8 mm, 1.46×	TOL 45.78 mm; module 55.5 mm, 32.74 g	5D‐PSF model + neural restoration
Chen et al. (2025) [46]	Double‐sided telecentric inspection	Yes	2 liquid lens groups	Multi‐element telecentric groups	−0.44× to −0.183×	275 mm system length	Refractive telecentric design
**This work**	Miniature meta‐liquid zoom imager	Yes	**2 EWOD liquid lenses**	**2 spherical lenses + 1 metalens**	**4–6 mm, 1.5**×	**15.5 mm TOL**	**Cross‐scale constrained optimization + metalens dispersion/aberration engineering**

*Note:* The comparison focuses on system complexity, zoom capability, size, and correction strategy; because the application scenarios, apertures, sensors, and post‐processing pipelines differ substantially among these systems, the image‐quality metrics are not used for direct ranking.

## 3 Conclusion

In this study, a cross‐scale synergistic optimization framework is established for metasurface–liquid lens hybrid imaging systems to address the disconnection between macroscopic optical‐system optimization and microscopic physical constraints. The framework incorporates the voltage‐curvature equality constraints of liquid lenses, the local phase‐gradient/GD inequality constraints of the metasurface, and system‐level RMS spot, MTF, and longitudinal chromatic‐aberration merit functions into a unified optimization process. As a result, each zoom state satisfies not only the imaging‐performance requirement but also the actual actuation and manufacturability conditions of the devices.

Based on this method, a miniature visible‐band achromatic zoom system with a total optical length of 15.5 mm is designed. With two electrowetting liquid lenses, two spherical refractive lenses, and one achromatic metalens, the system achieves a 1.5× continuous zoom over a 4–6 mm focal‐length range and a 10° field of view. The design is further evaluated through constraint–convergence trajectories, tolerance analysis, etch‐height error analysis, purely refractive baseline comparison, and full‐system wave–optics simulation. The results show that the metalens acts as a dispersion and high‐order aberration modulator in this system. Multi‐zone GD folding and boundary–phase optimization mitigate the longitudinal chromatic aberration, ghost foci, and multi‐wavelength interference mismatch of the baseline design under manufacturable constraints.

The cost and boundary of the approach are also clarified. Because multi‐zone phase folding inevitably introduces phase discontinuities and higher diffraction orders, the optimized system still contains sidelobe and halo energy relative to the ideal continuous‐phase system. One reason is that the present dispersion engineering uses 55 annular zones, so only a finite number of zone‐level interference relations can be adjusted. A larger number of zones may provide finer control of the axial and multi‐wavelength interference distribution and may further reduce ghost foci and diffraction halo, although this would also tighten the phase‐gradient, GD, and fabrication requirements. The residual halo therefore limits raw contrast transfer and reveals a system‐level trade‐off among short total length, low liquid‐lens count, manufacturable metasurface dispersion control, and visible‐band achromaticity. Future work will verify the present numerical analysis through fabricated samples, measured MTF/PSF, and full electromagnetic re‐simulations of height‐error effects.

Overall, this work links the dynamic zooming capability of liquid lenses with the high‐degree‐of‐freedom dispersion and aberration modulation capability of metasurfaces within a single physically constrained optimization framework. It provides a practical route from “device superposition” toward “system‐level synergistic design” for biomedical imaging, adaptive optics, augmented‐reality displays, and other compact dynamic‐imaging scenarios.

## Author Contributions

**Zhuoxuan Fan:** writing – original draft, methodology, software, investigation, formal analysis. **Meng Zhang:** writing – review and editing, investigation. **Zihao Gan:** writing – review and editing, investigation. **Mengyue Chang:** investigation, validation. **Ce Zhang:** investigation, validation. **Zhaoyang Liu:** resources, validation, visualization. **Jianming Lv:** resources, visualization. **Kunsheng Xing:** resources, visualization. **Zhuoqun Hu:** data curation, resources, visualization. **Junbo Yang:** writing – review and editing, resources, supervision. **Huajie Hong:** conceptualization, project administration.

## Funding

The National Natural Science Foundation of China (62305386).

## Conflicts of Interest

The authors declare no conflicts of interest.

## Data Availability

The data that support the findings of this study are available from the corresponding author upon reasonable request.

## Appendix A Physical Constraint Model of the Hybrid Meta‐Optical System

In the cross‐scale synergistic optimization process, the solution space must be strictly confined within boundary conditions imposed by fundamental physical laws, material properties, and micro‐nanofabrication processes.

For the liquid lens actuation module, its physical response is governed by the electrowetting dynamics model. The actual liquid–liquid interface curvature, $\kappa_{\text{liquid}}$, must satisfy the voltage–curvature mapping relation derived from the Young–Lippmann equation:

(A1)
$$h_{j}\left(x\right)={\kappa_{\text{liquid}}}_{j}\left(x\right)-\left(\kappa_{0}+\frac{\varepsilon_{r}\varepsilon_{0}}{2\gamma_{ci}dR}{U_{j}}^{2}\right)=0$$

where ${\kappa_{\text{liquid}}}_{j}\left(x\right)$ represents the target curvature determined by the system optimization variables x for the j‐th zoom configuration. In practice, zooming is realized by adjusting the drive voltage $U_{j}$. This study utilizes a commercial liquid lens (model: A25‐HX‐D0, Corning Varioptic), whose precise voltage‐curvature response curve and voltage‐fluid layer thickness relationship have been numerically modeled based on factory calibration data.

For the metasurface device, the phase distribution is characterized in OpticStudio using a user defined surface (UDS) model defined by even‐order polynomials. To ensure physical manufacturability, the local numerical aperture, NA=|∇ϕ|/k, determined by the local phase gradient, must be lower than the diffraction limit, $NA_{\max}=\lambda /\left(PN\right)$, imposed by the sub‐wavelength structural period P, where N>2 is the minimum sampling factor required by the Nyquist sampling theorem [47]. Combining this with Equation (3), the local fabrication constraint can be formulated as inequality constraint $g_{1}$:

(A2)
$$g_{1}\left(r,\lambda ;x\right)=\left|\frac{2\pi }{\lambda }\sum_{i=1}^{N_{i}}2iA_{i}r^{2i-1}\right|-\frac{2\pi }{PN}\leq 0,\;\forall r\in \left[0,R_{\max}\right],\forall \lambda \in \Lambda$$

where $N_{i}$ is the polynomial order, $R_{\max}$ is the effective radius of the metasurface, and Λ is the operating waveband. Furthermore, although the multi‐zone dispersion engineering strategy introduced in this study mitigates the absolute demand for group delay (GD) via phase wrapping, this does not imply that GD can increase indefinitely. Excessive GD requirements would necessitate a drastic increase in the number of zones, leading to a drop in diffraction efficiency due to scattering at zone boundaries, thereby weakening the metasurface's advantage over traditional diffractive elements in stray light suppression [48]. Therefore, a maximum group delay constraint, $g_{2}$, must be introduced:

(A3)
$$g_{2}\left(r;x\right)=\frac{1}{c}\left|\sum_{i=1}^{N_{i}}A_{i}r^{2i}\right|-GD_{\max}\left(\lambda \right)\leq 0,\;\forall r\in \left[0,R_{\max}\right],\forall \lambda \in \Lambda$$

where c is the speed of light in vacuum. This constraint directly limits the aspect ratio requirements of the meta‐atoms and indirectly controls the partition density of phase folding. Based on preliminary experimental results, we set $GD_{\max}=130$ fs. The optimization results show that the actual maximum group delay of the metalens is 117.5 fs (as shown in Figure 6c), satisfying the physical feasibility requirement.

For conventional glass lenses, standard manufacturing constraints such as the center thickness, edge thickness, and radii of curvature are controlled via built‐in operands in OpticStudio and are not detailed here.

At the algorithmic implementation level, all the aforementioned physical constraints are converted into an unconstrained optimization problem via the penalty function method Equation (4). Specifically, user defined operands (UDOs) were written using OpticStudio's macro language (ZPLM) and C# interface to realize real‐time calculation and feedback of these complex physical models during optimization.

## Appendix B Optical Prescription of the Hybrid System

The detailed structural parameters of the optimized meta‐liquid hybrid system are listed in Table A1. The internal structure of the liquid lens module includes the cap glass, conductive phase, oil bodycone, oil cylinder, bodycone windows, and necessary mechanical assembly gaps. The metasurface phase profile is described by even‐order aspheric polynomials, the coefficients of which are detailed in Table A2.

**TABLE A1:** Optical prescription of the hybrid meta‐liquid system.

Surf.	Component/Type	Curvature $\left({mm}^{-1}\right)$	Thickness (mm)	Material	Conic constant
OBJ	Object plane	0.0000	∞	—	—
2	Mechanical gap	0.0000	0.8000	Air	—
3	Air gap	0.0000	0.1000	Air	—
4	L1: Cap glass	0.0000	0.3323	D263TECO	—
5	L1: Conductive phase	0.0000	0.4741	PC282B	—
6	L1: Oil bodycone	−0.1239	0.1259	H575A	—
7	L1: Oil cylinder	0.0000	0.1000	H575A	—
8	L1: Bodycone window	0.0000	0.3000	D263TECO	—
9	Mechanical gap	0.0000	0.7998	Air	—
10	S1: Front surface	0.1667	0.2996	N‐BK7	3.4366
11	S1: Back surface	0.2702	4.8373	—	1.7299
12	Aperture stop	0.0000	0.2353	Air	—
13	S2: Front surface	−0.1173	0.4243	S‐FPL53	99.9922
14	S2: Back surface	−0.2538	0.1966	—	17.7671
17	Metasurface^a^	0.0000	0.3000	N‐BK7	—
18	Substrate back	0.0000	0.4953	—	—
19	Mechanical gap	0.0000	0.8000	Air	—
20	Air gap	0.0000	0.1000	Air	—
21	L2: Cap glass	0.0000	0.3323	D263TECO	—
22	L2: Conductive phase	0.0000	0.3322	PC282B	—
23	L2: Oil bodycone	0.2211	0.2678	H575A	—
24	L2: Oil cylinder	0.0000	0.1000	H575A	—
25	L2: Bodycone window	0.0000	0.3000	D263TECO	—
26	Mechanical gap	0.0000	0.8000	Air	—
27	Back focal distance	0.0000	2.3853	Air	—
28	Sensor cover glass	0.0000	0.5000	N‐BK7	—
IMG	Image plane	0.0000	—	—	—

^a^ Surface 17 represents the metasurface phase profile. See Table A2 for coefficients.

**TABLE A2:** Optimized phase polynomial coefficients for the metasurface (surface 17).

Term $\left(r^{n}\right)$	Coefficient $\left(A_{i}\right)$	Term $\left(r^{n}\right)$	Coefficient $\left(A_{i}\right)$
$r^{2}$	$-6.835\times 10^{-2}$	$r^{18}$	$1.063\times 10^{0}$
$r^{4}$	$-2.706\times 10^{-2}$	$r^{20}$	$3.397\times 10^{0}$
$r^{6}$	$3.438\times 10^{-1}$	$r^{22}$	$9.259\times 10^{0}$
$r^{8}$	$-1.515\times 10^{0}$	$r^{24}$	$3.143\times 10^{1}$
$r^{10}$	$2.870\times 10^{0}$	$r^{26}$	$-4.241\times 10^{1}$
$r^{12}$	$1.572\times 10^{-1}$	$r^{28}$	$-1.802\times 10^{2}$
$r^{14}$	$-6.325\times 10^{0}$	$r^{30}$	$2.803\times 10^{2}$
$r^{16}$	$-9.428\times 10^{0}$	$r^{32}$	$-9.516\times 10^{1}$

## Appendix C Algorithm for Multi‐Zone Dispersion Engineered Metalens

In the optimization of the achromatic metalens based on multi‐zone dispersion engineering, considering the design requirement for polarization insensitivity, this study adopts scalar diffraction theory—specifically, the angular spectrum method (ASM)—for optical field propagation calculations. The complex amplitude distribution U(x,y,z) after propagating a distance z in free space can be expressed as follows:

(A4)
$$U\left(x,y,z\right)=F^{-1}\left\{F\left[U\left(x,y,0\right)\right]\cdot \exp\left(jkz\sqrt{1-\alpha_{x}^{2}-\alpha_{y}^{2}}\right)\right\}$$

where $\alpha_{x}=\lambda f_{x}$ and $\alpha_{y}=\lambda f_{y}$ are the direction cosines, k=2π/λ is the wavenumber, and F and $F^{-1}$ denote the two‐dimensional Fourier transform and its inverse, respectively. To ensure calculation accuracy and eliminate spectrum aliasing, the sampling interval in the simulation space was set to dx=dy=0.16μm, and a 4× zero‐padding strategy was employed. The algorithm was written based on the CUDA architecture and implemented with fully parallel acceleration on an NVIDIA A800 GPU server.

When optimizing the phase jumps at the boundaries of each zone, the objective is to maximize the match between the weighted image–plane optical field M and the ideal achromatic field I. We define the weighted optical field as $M=U_{o}◦W$, where $U_{o}$ is the actual propagated field, W is the spatial Gaussian weight matrix, and ◦ denotes the Hadamard product. The merit function employs a generalized cosine similarity defined based on the Frobenius inner product. For a broadband optical field containing K discrete wavelengths, the similarity function is defined as follows:

(A5)
$$S=\frac{\left|{\langle M,I\rangle }_{F}\right|}{\|M{\|}_{F}\|I{\|}_{F}}=\frac{\left|\sum_{\lambda \in K}\sum_{x,y}\bar{M\left(x,y,\lambda \right)}\cdot I\left(x,y,\lambda \right)\right|}{\sqrt{\sum |M{|}^{2}}\cdot \sqrt{\sum |I{|}^{2}}}$$

where $\|\cdot {\|}_{F}$ denotes the Frobenius norm, and the overbar $\bar{\cdot }$ denotes the complex conjugate. This metric constrains not only the intensity distribution (amplitude) but, crucially, also introduces a strict phase‐matching constraint. This full complex‐amplitude optimization strategy effectively suppresses destructive interference caused by dispersion near the focal point, thereby significantly improving axial chromatic aberration and the distribution of high‐order diffraction sidelobes.
